# A Randomized Trial Evaluating the Effect of Bundled Preoperative Skin and Vaginal Cleaning Protocols on Surgical Site Infections Following Cesarean Delivery

**DOI:** 10.7759/cureus.95831

**Published:** 2025-10-31

**Authors:** Shivangi Ghildiyal, Savita Somalwar, Anuja Bhalerao, Sheela Jain

**Affiliations:** 1 Department of Obstetrics and Gynaecology, Narendra Kumar Prasadrao (NKP) Salve Institute of Medical Sciences and Research Centre, Nagpur, IND

**Keywords:** cesarean section, preoperative care, randomized controlled trial, surgical care bundle, surgical site infection

## Abstract

Background: Surgical site infections (SSIs) following cesarean section (CS) contribute to prolonged hospital stays, increased healthcare costs, maternal morbidity, and psychological stress. Most surgical safety bundles have been shown to reduce SSIs, but the impact of incorporating preoperative skin preparation and vaginal cleaning into these bundles has not been comprehensively assessed in India.

Aim: This study aimed to evaluate the effect of incorporating preoperative skin preparation and vaginal cleaning into standard care bundles for the prevention of SSIs following CS.

Methods: This randomized controlled trial was conducted at a tertiary care hospital in central India. A total of 170 women undergoing elective or emergency CS were randomly assigned to two groups. In group A, before the CS, parts were prepared by hair clipping instead of shaving. Vaginal cleaning was performed with a 0.25% chlorhexidine solution. Group B received the standard bundle without additional preoperative care and vaginal cleaning. Intraoperative and postoperative care were uniform across groups. The primary outcome was the incidence of SSI within 30 days; secondary outcomes included wound condition at Day 9, microbiological profile, and duration of hospital stay.

Results: The overall SSI incidence was 8.8%. Group A had a lower infection rate (5.9%) than Group B (11.8%) (p = 0.0159). Deep SSIs were markedly reduced in the intervention group, 1 (1.2%) vs. 9 (10.6%). On Day 9, healthy wound healing was seen in 80 (94.1%) of Group A compared to 75 (88.2%) of Group B. Pyrexia, wound gape, and resuturing were more frequent in controls. Culture reports supported reduced colonization in the intervention group. The mean hospital stay was significantly shorter in Group A (9.2 ± 2.6 days) compared with Group B (10.1 ± 1.0 days; p = 0.0133).

Conclusion: Addition of preoperative skin preparation and vaginal cleaning to routine surgical bundles significantly decreases SSI rates, particularly deep infections, and reduces the length of stay after CS. These low‑cost, easily adoptable interventions are especially valuable in resource‑limited settings. Wider institutional adoption and regular monitoring are recommended to optimize maternal surgical outcomes.

## Introduction

Cesarean section (CS) is an essential surgical intervention in contemporary obstetric care, often safeguarding maternal and neonatal well-being when vaginal delivery poses excessive risk. However, with the increasing global reliance on CS, 32.1% in the United States [1] and nearly 21.5% in India as per the latest National Family Health Survey (NFHS) data [2], the associated risk of postoperative complications, especially surgical site infection (SSI), has become a major concern for healthcare systems worldwide.

SSIs after CS not only prolong healthcare costs and hospital stay but also negatively impact maternal recovery and psychological well-being [3]. The complex pathogenesis of SSI involves multifactorial risk determinants, of which intraoperative and perioperative processes are critically modifiable [4].

SSIs are infections that occur at or near the surgical incision within 30 days postoperatively. They are categorized into superficial incisional, deep incisional, and organ/space infections. Superficial infections involve only the skin and subcutaneous tissue, while deep infections affect the deeper soft tissues, including muscle and fascia. Organ/space infections, the most severe form, involve structures beyond the incision, such as the endometrium or peritoneal cavity [5,6].

Human skin flora, such as *Staphylococcus aureus*, and more resistant forms, such as methicillin-resistant *Staphylococcus aureus *(MRSA), remain common microbial culprits [7]. Equally, pathogens from the vaginal microenvironment, especially pathogens like *Escherichia coli* and Group B Streptococcus, can ascend or contaminate operative sites, particularly when amniotic membranes are ruptured for extended periods or in cases involving frequent per-vaginal (PV) examinations.

The risk of SSIs is heightened in CS due to factors such as prolonged labor, membrane rupture, obesity, diabetes, and inadequate antiseptic measures. The consequences of SSIs include increased antibiotic use, delayed wound healing, readmissions, and, in extreme cases, hysterectomy or maternal mortality. Given these substantial impacts, it is imperative to optimize infection-prevention strategies for women undergoing CS.

The World Health Organization (WHO) and various quality-improving consortia advocate the use of structured, evidence-based “bundles” of surgical safety measures. Such bundles typically include antibiotic prophylaxis, aseptic surgical field preparation, hair clipping (rather than shaving), and intraoperative thermal maintenance. While these interventions have proven value, emerging data support the integration of expanded elements such as preoperative alcohol-based skin antisepsis and thorough vaginal cleaning. Studies indicate that such targeted steps further reduce the microbial burden and ultimately the risk of SSI [8-10].

Despite the well-documented rise in CS rates in India, now at a national average of 21.5%, there remains limited research evaluating low-cost, comprehensive infection prevention bundles adapted to local settings. In India’s mixed-resource healthcare environment, the impact of combining preoperative skin preparation with vaginal cleaning as part of a structured care bundle for cesarean deliveries has not been systematically studied.

This randomized controlled trial aimed to evaluate the effect of incorporating preoperative skin preparation and vaginal cleaning into standard care bundles for the prevention of SSIs following CS.

## Materials and methods

Study design

This prospective, randomized controlled study was conducted at the Department of Obstetrics and Gynecology in a tertiary care hospital of central India from August 2023 to July 2025. Institutional ethical clearance was obtained prior to patient enrollment. Women presenting for elective or emergency CS and meeting inclusion/exclusion criteria were recruited after obtaining informed, written consent.

Participant selection

The study included pregnant women aged 18-40 years undergoing emergency or elective CS during the specified period. Exclusion criteria were as follows: known immunodeficiency, ongoing systemic infection at the time of surgery, documented allergy to antiseptic agents, and those declining to participate. All participant data were anonymized and stored securely.

Randomization and interventions

The sample size was calculated based on the anticipated difference in postoperative SSI proportions between the two groups. Assumptions were derived from a previous study by Ahmed et al. [11], in which the SSI incidence was 24.4% in Group 1 and 8.6% in Group 2, yielding an effect size of 15.8%. Considering a power of 80% and a two-sided α error of 5%, the sample size of 85 participants in each group was determined.

Participants were randomized in equal proportions to two groups using computerized block randomization to ensure balanced group sizes throughout the study. The randomization sequence was concealed and only revealed immediately before surgery to prevent selection bias. In group A (intervention) and group B (control), the preoperative surgical bundle that followed was different. In both groups, the intraoperative and postoperative bundles were the same.

Group A (intervention)

In the intervention group, before CS, parts were prepared by hair clipping instead of shaving. Vaginal cleaning was performed with a 0.25% chlorhexidine solution. Skin preparation was performed with 0.25% chlorhexidine gluconate solution, followed by povidone-iodine. Injection (Inj.) cefuroxime 1.5 g and Inj. metronidazole 100 mL were given 30-60 minutes before skin incision. The surgeon ensured all nine steps of hand washing with soap and water to avoid infection, for a minimum of two to three minutes.

Group B (control)

A standard surgical care bundle was implemented without any preoperative preparation or vaginal cleaning. Before CS, parts were prepared by shaving. Injectable antibiotics were given. The operating surgeon maintained hand hygiene. Hand hygiene compliance was maintained by regular staff training and direct observation by infection control personnel. Adherence was reinforced through routine feedback and monitoring.

Intraoperative steps were standardized for both groups. Skin preparation was done sequentially with chlorhexidine, povidone-iodine, spirit, and isopropanol and benzalkonium. Throughout the procedure, normothermia and normoglycemia were maintained. The surgeon changed gloves after closure of the viscera and muscles and used a fresh pair for skin closure to minimize infection risk.

Postoperatively, all patients received intravenous antibiotics (cefuroxime 1.5 g twice daily and metronidazole 100 mL thrice daily) for 48 hours. Dressings were inspected twice daily for soakage; if present, wound swabs were sent for culture and sensitivity testing, and antibiotics were adjusted accordingly. In the absence of soakage, the dressing was changed on Day 4, while sutures were removed on Day 9 under aseptic conditions. Patients were monitored for discharge characteristics, local signs of infection, abscess formation, wound induration, misapproximation, or gape. After discharge, follow‑ups were scheduled at 15 days and one month, with continued monitoring for infection or wound-related complaints.

Data collection

Baseline variables included maternal age, body mass index (BMI), parity, gravida, hemoglobin-based anemia status, booking status, and the number of antenatal visits. Preoperative assessments documented PV examinations, indication for cesarean delivery, procedure type (elective vs. emergency), intraoperative events, and operative duration. Postoperative outcomes were assessed systematically, with surveillance for SSIs and wound status on Days 4 and 9, including predefined criteria for erythema, discharge, swelling, pyrexia, induration, wound gape, or abscess. Where clinically indicated, wound swabs were obtained for culture and sensitivity testing. Secondary outcomes included duration of postoperative hospital stay, 30‑day follow‑up assessment, and readmission rates.

Statistical analysis

Descriptive statistics were computed, and baseline characteristics were compared using t-tests and chi-square tests as appropriate. p values of <0.05 were deemed significant.

## Results

The age distribution was comparable between the two groups, with the largest proportion of participants in both cohorts belonging to the 26-30 years category: group A, 32 (37.6%), and group B, 33 (38.8%). Patients aged 22-25 years accounted for one‑third of the total study population, while only seven (4.1%) were above 35 years. The intergroup difference in age was not statistically significant (p = 0.590). The mean age was 26.18 ± 3.97 years in Group A and 27.07 ± 4.39 years in Group B (p = 0.165).

Similarly, BMI categories were evenly distributed across groups. Overweight women (23.0-24.9) formed the majority (60, 37.1%), followed by obesity I (51, 30.0%) and the normal BMI range (55, 32.4%), with very few participants underweight (1, 0.6%). No statistical difference was observed in BMI distribution (p = 0.720). The mean BMI was nearly identical across groups (23.79 ± 2.17 vs. 23.80 ± 2.18; p = 0.975).

Obstetric profiles were also balanced between the groups. Nearly half of the women were primigravida (80, 47.1%), while 52 (30.6%) had two pregnancies and 38 (22.4%) were multigravida. Parity distribution showed 100 (58.8%) nullipara, 61 (35.9%) primipara, and only nine (5.3%) with parity greater than or equal to two, without significant group differences (p > 0.05). Registration status revealed that 104 (61.2%) were booked, 54 (31.8%) registered, and 12 (7.1%) unbooked; group differences were not statistically significant (p = 0.171). Most participants (139, 81.8%) had greater than or equal to four antenatal visits, again with no significant difference between the groups (p = 0.551) (Table 1).

**Table 1:** Baseline variables of two study groups NS: not significant, BMI: body mass index, ANC: antenatal care

Variables		Group A (n = 85)	Group B (n = 85)	Total (n = 170)	Chi-square test
Age group	18-21 years	7 (8.2%)	6 (7.1%)	13 (7.6%)	χ²(4) = 2.81, p = 0.590, NS
	22-25 years	32 (37.6%)	25 (29.4%)	57 (33.5%)	
	26-30 years	32 (37.6%)	33 (38.8%)	65 (38.2%)	
	31-35 years	12 (14.1%)	16 (18.8%)	28 (16.5%)	
	>35 years	2 (2.4%)	5 (5.9%)	7 (4.1%)	
BMI category	Underweight (<18.5)	0 (0%)	1 (1.2%)	1 (0.6%)	χ²(3) = 1.34, p = 0.720, NS
	Normal (18.5-22.9)	28 (32.9%)	27 (31.8%)	55 (32.4%)	
	Overweight (23.0-24.9)	33 (38.8%)	30 (35.3%)	63 (37.1%)	
	Obesity I (25.0-29.99)	24 (28.2%)	27 (31.8%)	51 (30.0%)	
Gravida	Primigravida	41 (48.2%)	39 (45.9%)	80 (47.1%)	χ²(2) = 0.46, p = 0.793, NS
	Two gravidas	24 (28.2%)	28 (32.9%)	52 (30.6%)	
	Multigravida	20 (23.5%)	18 (21.2%)	38 (22.4%)	
Parity	Nulliparous	48 (56.5%)	52 (61.2%)	100 (58.8%)	χ²(2) = 1.18, p = 0.555, NS
	Primipara	31 (36.5%)	30 (35.3%)	61 (35.9%)	
	≥2	6 (7.1%)	3 (3.5%)	9 (5.3%)	
Registration status	Registered	22 (25.9%)	32 (37.6%)	54 (31.8%)	χ^2^(2) = 3.53, p = 0.171
	Booked	55 (64.7%)	49 (57.6%)	104 (61.2%)	
	Unbooked	8 (9.4%)	4 (4.7%)	12 (7.1%)	
ANC visits	<4 visits	14 (16.5%)	17 (20.0%)	31 (18.2%)	χ^2^(1) = 0.36, p = 0.551
	≥4 visits	71 (83.5%)	68 (80.0%)	139 (81.8%)	

The prevalence of anemia was similar across groups, affecting 66 (38.8%) of the total study population, with no significant difference between Group A (34, 40.0%) and Group B (32, 37.6%) (p = 0.753). In Group A, 27 women had mild anemia, while seven women had moderate anemia. In Group B, out of 32 anemic women, 22 had mild and 10 had moderate anemia. The remaining participants, accounting for 51 (60.0%) in Group A and 53 (62.4%) in Group B, did not have anemia. Hemoglobin levels were comparable as well, with Group A recording a mean of 11.26 ± 1.02 g/dL and Group B 11.02 ± 1.10 g/dL (p = 0.136).

Most participants (114, 67.1%) underwent fewer than four per‑vaginal (PV) examinations prior to surgery, with no significant intergroup variation (p = 0.514). Regarding the type of CS, 111 (65.3%) were performed electively, while 59 (34.7%) were emergency procedures, with distributions nearly identical between the two study arms (p = 0.872) (Table 2).

**Table 2:** Distribution of anemia, the number of PV examinations, and the type of CS PV: per-vaginal, CS: cesarean section

Variables		Group A (n = 85)	Group B (n = 85)	Total (n = 170)	Chi-square test
Anemia	Yes	34 (40.0%)	32 (37.6%)	66 (38.8%)	χ^2^(1) = 0.10, p = 0.753
	No	51 (60.0%)	53 (62.4%)	104 (61.2%)	
Number of PV examinations	≥4	26 (30.6%)	30 (35.3%)	56 (32.9%)	χ^2^(1) = 0.43, p = 0.514
	<4	59 (69.4%)	55 (64.7%)	114 (67.1%)	
Emergency or elective CS	Elective	55 (64.7%)	56 (65.9%)	111 (65.3%)	χ^2^(1) = 0.03, p = 0.872
	Emergency	30 (35.3%)	29 (34.1%)	59 (34.7%)	

The distribution of indications for CS was largely comparable between the two groups. A prior cesarean delivery with the patient not willing for a trial of labor was the most frequent indication (51, 30.0%), followed by hypertensive disorders of pregnancy (24, 14.1%), malpresentation (21, 12.4%), and fetal distress (21, 12.4%). Cephalopelvic disproportion accounted for 18 (10.6%) of cases, while failed induction contributed to 16 (9.4%) cases. Less common indications included bad obstetric history (3, 1.8%) and previous CS with scar tenderness (6, 3.5%). No meaningful intergroup variation was observed across these indications (Table 3). The overall incidence of SSI was 8.8%, with a significantly higher occurrence in Group B (11.8%) than in Group A (5.9%) (p = 0.0159). Superficial SSI was identified in 2.9% of cases (Group A: 4.7%, Group B: 1.2%), while deep SSI was more frequent in Group B (10.6%) than in Group A (1.2%) (p = 0.159).

**Table 3:** Distribution of indication for CS between two study groups CS: cesarean section

Indication for CS	Group A (n = 85)	Group B (n = 85)	Total (n = 170)
Bad obstetric history	2 (2.4%)	1 (1.2%)	3 (1.8%)
Cephalopelvic disproportion	9 (10.6%)	9 (10.6%)	18 (10.6%)
Fetal distress	11 (12.9%)	10 (11.8%)	21 (12.4%)
Failed induction	4 (4.7%)	12 (14.1%)	16 (9.4%)
Hypertensive disorder of pregnancy	12 (14.1%)	12 (14.1%)	24 (14.1%)
Malpresentation	12 (14.1%)	9 (10.6%)	21 (12.4%)
Pre-CS not willing for trial of labor	25 (29.4%)	26 (30.6%)	51 (30.0%)
Previous CS with scar tenderness	5 (5.9%)	1 (1.2%)	6 (3.5%)

Among clinical signs of SSI, rates of tenderness, redness, swelling, heat, and induration were higher in Group B, although these differences were not statistically significant (p > 0.05). Pyrexia, a marker suggestive of deep SSI, was observed exclusively in Group B (6, 7.1%), reaching statistical significance (p = 0.0285). Wound gape was more commonly reported in Group B (7, 8.2%) than in Group A (1, 1.2%), showing a trend toward significance (p = 0.0639). Abscess formation was rare and did not differ significantly between the groups (Table 4).

**Table 4:** Distribution of SSIs and signs of SSIs in two groups SSI: surgical site infection

Variable		Group A (n = 85)	Group B (n = 85)	Total (n = 170)	p values
SSI status in the two study groups	No SSI	80 (94.12%)	75 (88.2%)	155 (91.2%)	p = 0.01589: significant
	Superficial SSI	4 (4.7%)	1 (1.2%)	5 (2.9%)	
	Deep SSI	1 (1.2%)	9 (10.6%)	10 (5.9%)	
Signs of SSI	Tenderness	2 (2.35%)	6 (7.1%)	8 (4.7%)	χ^2^ = 1.18, p = 0.277
	Localized swelling	3 (3.51%)	3 (3.51%)	11 (6.47%)	χ^2^ = 0.17, p = 0.67
	Redness	2 (2.35%)	7 (8.23%)	9 (5.29%)	χ^2^ = 1.82, p = 0.17
	Heat	3 (3.51%)	8 (9.41%)	11 (6.47%)	χ^2^ = 1.55, p = 0.21
	Induration at the surgical site	5 (5.9%)	8 (9.41%)	13 (7.65%)	χ^2^ = 0.33, p = 0.56
Sign of deep SSI	Pyrexia	0 (0.0%)	6 (7.1%)	6 (3.53%)	Fisher's exact test, p = 0.0285, significant
	Abscess	0 (0.0%)	2 (2.35%)	2 (1.2%)	Fisher's exact test, p = 0.497, not significant
	Wound gap	1 (1.2%)	7 (8.2%)	8 (4.7%)	Fisher's exact test, p = 0.0639, not significant

Microbiology

Of the 11 participants whose wounds were cultured (clinical suspicion of SSI), three (75%) of intervention group samples yielded no growth; the only pathogen seen was Klebsiella. In sharp contrast, all control group swabs (8, 100%) were positive: MRSA (most frequent), Klebsiella, and *E. coli*. This aligns with the hypothesized mechanism by which preoperative antisepsis reduces both overall colonization and the proportion of multidrug-resistant pathogens.

On day 9 postoperatively, most women in both groups demonstrated healthy wound healing (155, 91.2%), with no statistically significant difference between Group A (80, 94.1%) and Group B (75, 88.2%) (p = 0.279). Unhealthy wounds were observed in 15 cases (8.8%) overall.

The institution's protocol was to discharge CS patients on post-CS day 9, after suture removal. The practice is based on the patient's willingness to remain in the hospital and take adequate rest, with good wound care provided. In terms of hospital stay, most patients (155, 91.2%) were discharged on the ninth day after surgery, though the distribution differed significantly between groups (p = 0.043). Prolonged hospitalization beyond 15 days was more common in Group B (8, 9.4%) compared to Group A (1, 1.2%), while intermediate stays (10-15 days) were infrequent in both cohorts (Table 5).

**Table 5:** Wound status on Day 9 and the number of hospital stays

Variables		Group A (n = 85)	Group B (n = 85)	Total (n = 170)	Chi-square test
Wound status (Day 9 postoperative)	Unhealthy	5 (5.9%)	10 (11.8%)	15 (8.82%)	χ^2^ = 1.16, p = 0.279
	Healthy	80 (94.1%)	75 (88.2%)	155 (91.18%)	
Number of days of hospital stay	<10 days	80 (94.1%)	75 (88.2%)	155 (91.2%)	χ^2^ = 6.27, p = 0.043,
	10-15 days	4 (4.7%)	2 (2.4%)	6 (3.5%)	
	>15 days	1 (1.2%)	8 (9.4%)	9 (5.3%)	

The mean hospital stay was shorter in the intervention arm by nearly a full day. Group A had a shorter mean hospital stay (9.21 ± 2.67 days) than Group B (10.17 ± 1.08 days), and this difference was statistically significant (p = 0.0133), indicating a reduced financial and social burden.

Comparing elective and emergency CS, SSI rates were not significantly different (10.2% vs. 8.1%). Other perioperative factors, such as the number of PV examinations and gravidity, did not significantly affect SSI incidence, reflecting the balanced randomization.

## Discussion

CS rates in India have been steadily rising, reflecting broader global trends driven by increased access to healthcare facilities, changing maternal demographics, and evolving clinical practices. With the growing number of cesarean deliveries, SSIs have become a significant concern, as they can lead to prolonged hospital stays, higher treatment costs, and increased maternal morbidity. The adoption of surgical care bundles, comprehensive protocols that combine evidence-based measures such as proper hand hygiene, timely antibiotic administration, optimal skin preparation, and standardized surgical techniques, has shown promise in reducing the incidence of SSIs.

The current randomized control trial was conducted in a tertiary care institute wherein patients undergoing CS were randomly subjected to a surgical bundle with or without preoperative skin preparation and vaginal cleaning (Group A and Group B, respectively).

Age

The current study found that most participants in both Group A and Group B (each n = 85) were 22-25 or 26-30 years old, accounting for over 70% of the study population. These findings reflect a common pattern in pregnancy cohorts, especially in India and similar settings, where the majority of pregnancies occur in women aged 20-30 years [12,13].

BMI

In our study, BMI was nearly identical between the groups, with means of 23.79 (SD = 2.17) for Group A and 23.8 (SD = 2.18) for Group B, showing no significant difference (p = 0.975). The BMI categories were also equivalent in both groups. Fifty-five women (32.4%) were of normal weight, while 63 (37%) were overweight. BMI categories were also similar in both groups (p = 0.72). Regarding BMI, the majority of women in similar studies had BMI in the normal or overweight range, with few falling into the obese category, mirroring trends observed in Indian studies using Asian-specific BMI criteria [12,14].

Gravida and parity status

In this study, almost half of the women were in their first pregnancy, 41 (48.2%) in Group A and 39 (45.9%) in Group B. Around one‑third were in their second pregnancy, while the remaining had three or more. The gravida status was similar across groups. Likewise, over half were nulliparous, about one‑third were primipara, and a small proportion had two or more previous births. Parity distribution also showed no significant difference (p = 0.555). The study reflects trends observed in the Indian obstetric literature, where most antenatal women are in their first or second pregnancy, while those with three or more pregnancies constitute a smaller group [2,15].

Registration status and antenatal care visits

Among the women, 104 (61.2%) were booked cases, 54 (31.8%) registered, and 12 (7.1%) unbooked, with no significant difference between the groups (p = 0.171). Regarding antenatal care visits, 139 (81.8%) had four or more visits, whereas 31 (18.2%) had fewer, which also showed no significant difference between the groups (p = 0.551).

Anemia

Anemia was found in 34 (40%) of Group A and 32 (37.6%) of Group B (p = 0.753). Most cases were mild (49 of 66, 74.2%), with the remaining moderate, and none were severe. The average hemoglobin was 11.26 g/dL in Group A and 11.02 g/dL in Group B, with no significant difference (p = 0.136). These results align with earlier reports. In India, NFHS-5 (2019-2021) estimates show 52%-54% of pregnant women are anemic [16]. Average hemoglobin in Indian studies typically ranges from 8.8 to 10.5 g/dL, particularly low in rural and socioeconomically disadvantaged groups, with some rural studies reporting prevalence as high as 81%-88% [17,18].

Number of PV examinations

In this study, following departmental protocol, unnecessary PV examinations were avoided. About two-thirds of participants in both groups had fewer than four PV examinations, and the number of examinations was comparable between the groups.

Frequent PV examinations are a key risk factor for SSI after CS. A meta-analysis of 19 studies showed a 3.8-fold increased risk (adjusted odds ratio, aOR = 3.80, 95% confidence interval, CI: 2.45-5.88), while more than five PV exams raised the risk further (relative risk = 3.25, 95% CI: 2.39-4.42; aOR = 2.52, 95% CI: 1.01-6.30) [19]. An Indian study also reported that 68% of SSI cases followed repeated PV examinations, highlighting the need to minimize their frequency [20].

In tertiary care hospitals, a prior cesarean remains the chief indication, reported in about 30%-37% of cases, as repeat surgery is often preferred due to fear of uterine rupture and limited support for vaginal birth after cesarean [21,22]. Fetal distress is another major reason, contributing to 11%-20% of procedures, usually linked to inadequate oxygen supply to the fetus during labor [23].

In the current study, 111 (65.3%) participants underwent elective CS, while the remaining 34.7% underwent emergency CS. There was no difference between the two groups in elective or emergency CS rate.

Signs of inflammation

On Day 4 after surgery, soakage was noted in four cases (4.7%) of Group A and six cases (7.1%) of Group B, while discharge occurred in four cases (4.7%) and seven cases (8.2%) respectively; both differences were not significant. Inflammatory signs like tenderness, redness, swelling, local heat, and induration were more common in the control group, but without statistical significance. Regarding deep SSI, pyrexia was reported in six cases (7.1%) of Group B and none in Group A (p = 0.0285, significant). Wound dehiscence needing resuturing was lower in Group A (one case, 1.2% vs. seven cases, 8.2%, p = 0.0639), indicating notable clinical importance.

Day 4 wound soakage or discharge is an early indicator of SSI. In a Pune study of over 1,200 CS cases, Mhaske et al. noted that purulent discharge was the most common finding, observed in 26.7% of infections, often accompanied by wound dehiscence, pain, or induration. SSIs usually appeared within 5 ± 2 days after surgery, most often first noticed between Days 4 and 7 [24]. Similarly, a Rajasthan hospital study reported serosanguinous or purulent discharge in about 22% of cesarean wounds with poor healing, mostly within the first postoperative week [25]. In Hyderabad, Basany et al. followed 2,015 CS cases and found a 4.6% SSI rate, with most detected in the first week. Common features included purulent discharge, soakage, pain, and tenderness [26].

Culture report

In this study, wound swabs were tested from 11 patients with suspected infection: four in Group A and seven in Group B. In Group A, three cases (75%) showed no bacterial growth, while one case (25%) grew Klebsiella spp. In contrast, all swabs in Group B were positive, with MRSA being the most common (four of seven cases, 57.1%), followed by Klebsiella spp. (two cases, 28.6%) and *E. coli* (one case, 14.3%). These findings indicate not only fewer infections but also less-aggressive pathogens in the bundle group. Previous research also highlights *S. aureus* as a frequent cause of infection, with *Enterococcus faecalis* and Staphylococcus species commonly isolated. Prolonged surgery and anemia were noted as significant risk factors, and MRSA emerged as the leading pathogen, including in a Belagavi study where it was the most frequently cultured organism across both bundle and standard care groups.

Incidence of SSIs

In this study, SSIs occurred in 8.8% (15/170) of cases, with a notable difference between the groups. Group A, which received a surgical care bundle including vaginal cleaning and hair clipping, had a 5.9% (five cases) SSI rate, mostly superficial (four out of five cases). In contrast, the group without these measures had 10 SSIs, with nine being deep infections. The reduction in deep SSIs in the bundle group was statistically significant (p = 0.01589).

Basany et al., in a study conducted in India, reported an overall SSI rate of 4.6%, mostly superficial. A WHO-recommended single-dose antibiotic protocol was used in a tertiary setting [26]. As per the Centers for Disease Control and Prevention (CDC)/US Benchmark, SSI rates are typically 3%-5% with rare deep infections, reflecting gold-standard bundles and high compliance in high-income healthcare [5,6].

SSI reduced from 19.4% to 9.8% with additional interventions (vaginal betadine cleaning, chlorhexidine scrub, azithromycin with ceftriaxone, and earlier dressing change). However, deep SSI remained high (35.7%) in both groups [27].

A bundle of pre-, intra-, and post-op measures reduced SSI by half (from 19.4% to 9.8%), with a significant improvement (p = 0.02). At 48 hours, all intervention wounds were healthy, in contrast to only 80% of wounds in the control group [28].

In the current study, the overall SSI was 8.8%. Without skin preparation and vaginal cleaning, the rate was 11.8% (with deep SSIs at 10.6%). When these steps were implemented, the overall rate was 5.9%, with deep SSIs at 1.2%. Alcohol-based prep and vaginal cleaning significantly reduced deep SSI.

Hospital stay

As per hospital protocol, CS patients are discharged on the ninth day after suture removal. On this day, wound healing was healthy in 80 cases (94.1%) of Group A and 75 cases (88.2%) of Group B (p = 0.279). Comparable Day 9 data are scarce, but a Chandigarh study reported that SSIs were usually detected after the eighth postoperative day, with 3.49% of wounds being unhealthy by Day 9 [29].

Hospital stay was shorter in Group A, where the surgical bundle, including skin preparation and vaginal cleaning, was applied. The mean duration was 9.21 ± 2.67 days in Group A vs. 10.17 ± 1.08 days in Group B (p = 0.0133). Prolonged stays of 15 days were observed in only 1.2% (one case) of Group A, compared with 9.4% (eight cases) in Group B (p = 0.043), highlighting a reduced burden with fewer deep SSIs. Early discharge in Group A can reduce treatment costs by one extra day, which may be substantial in the private sector. In the Government sector, a reduced hospital stay may help accommodate another patient in need.

Multiple studies highlight that SSIs after CS markedly prolong hospital stay. In India, women without SSI stayed about five days, while those with infection remained for around 13.6 days [20,24]. A large international analysis similarly found longer stays in SSI cases (5.6 vs. 3.3 days) [30]. Data from Turkey and multicenter cohorts confirm that SSIs are a consistent predictor of extended admissions, delayed recovery, and higher readmission rates [21]. Global reviews, including CDC reports, estimate that SSIs add an average of 9-10 extra hospital days, reflecting both clinical and economic burdens [5].

Strengths

The study's main strengths are its randomized trial design, balanced baseline characteristics, and thorough data collection, which support a reliable comparison of interventions. Implementing simple, low-cost preoperative measures led to lower SSIs and shorter hospital stays, making these findings particularly relevant for resource-limited settings.

Limitation

The present study's findings should be interpreted in light of certain limitations. To begin with, it was conducted in a single tertiary hospital with a moderate sample size, which may limit how well the results can be applied to different institutions or broader populations. Also, despite attempts to standardize protocols, some variation in surgical practice and antibiotic use may have occurred, given the large teaching hospital setting. Further, while the study strengths lie in robust data collection and randomization, larger multicenter studies and longer term follow-up would be necessary to confirm these results and ensure their applicability in varied healthcare environments.

## Conclusions

This randomized controlled trial demonstrates that adding preoperative skin preparation with alcohol-based antiseptics and vaginal cleaning to the standard surgical bundle significantly lowers SSIs after CS, particularly reducing the incidence of deep infections. The intervention group also showed shorter hospital stays and fewer wound-related complications, reflecting both clinical and economic benefits. These findings support integrating simple, low-cost measures into existing bundles, especially in resource-limited settings, to improve maternal outcomes. Broader implementation at institutional and policy levels, along with regular staff training and protocol monitoring, is required to implement such bundles reliably.
